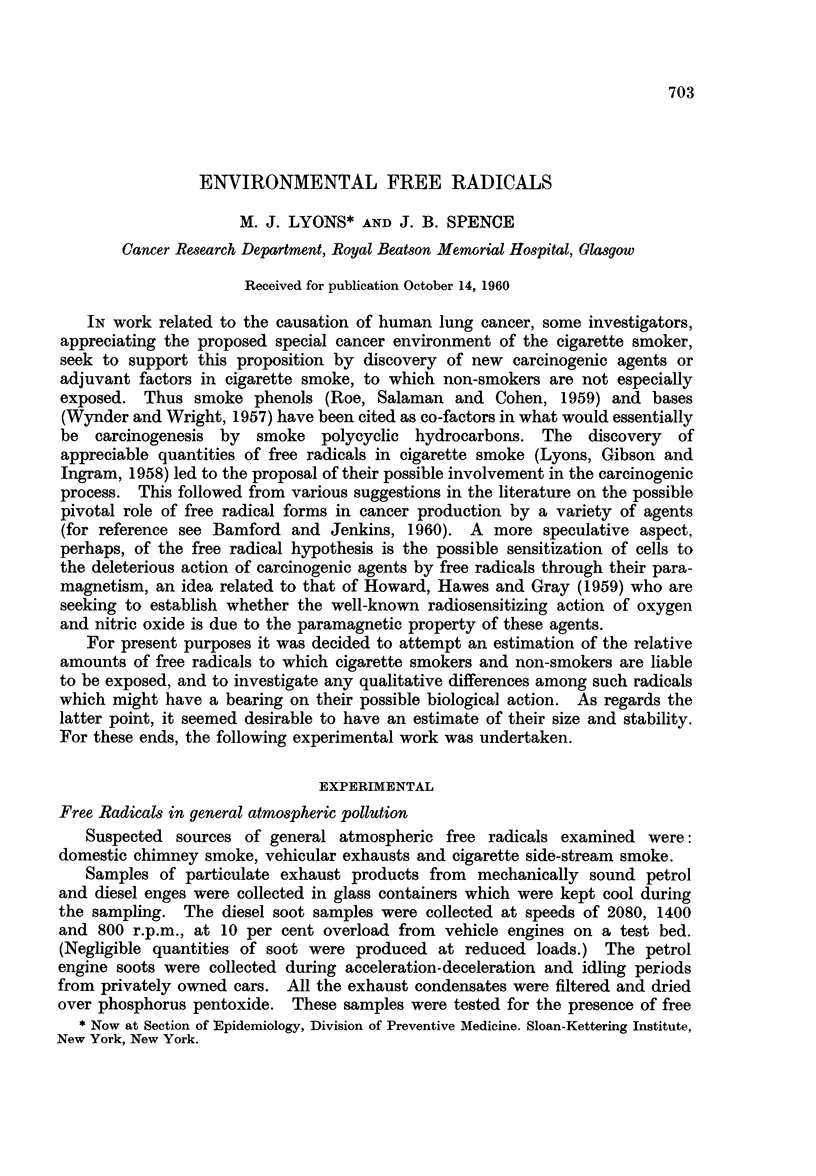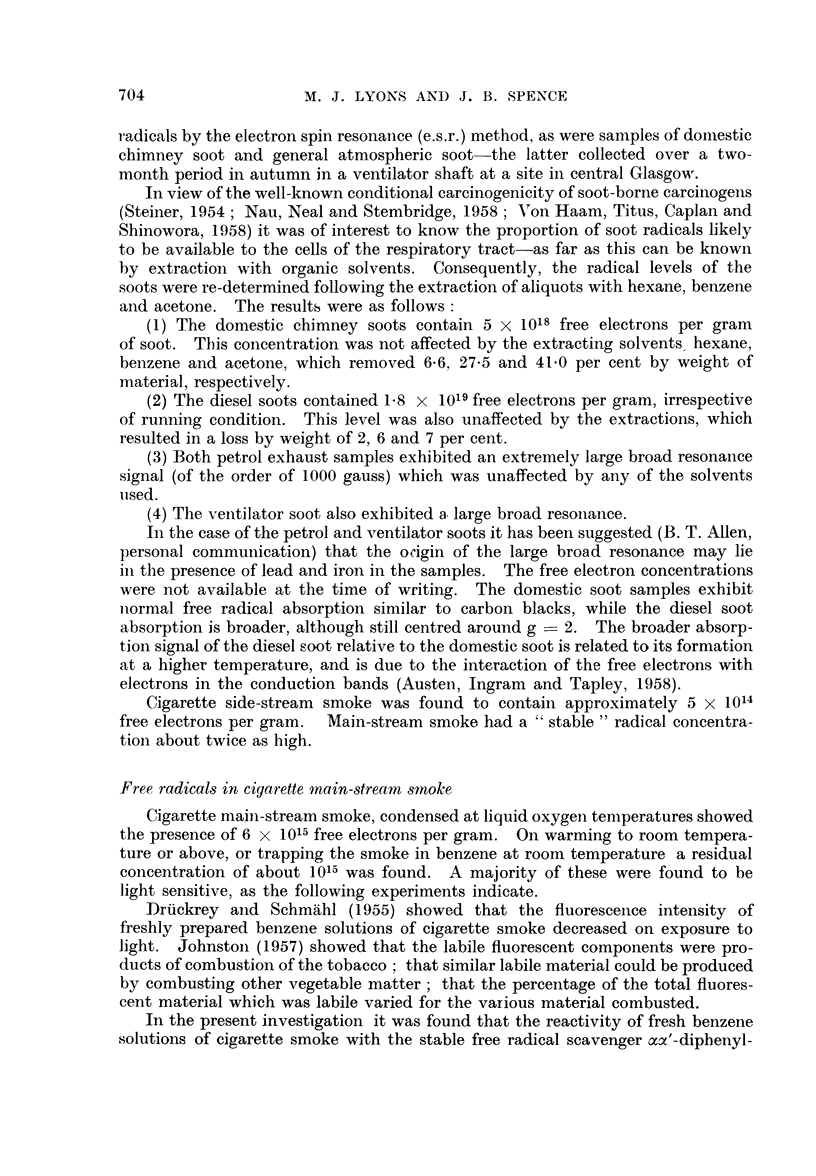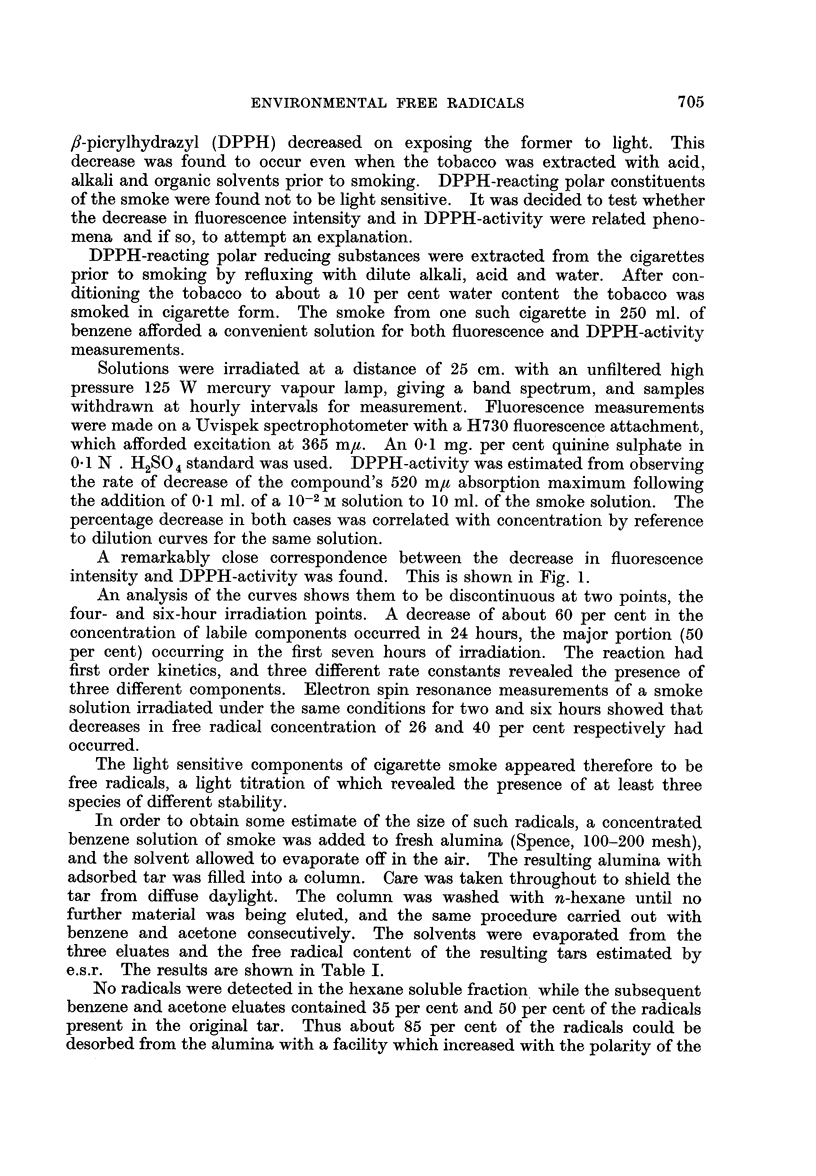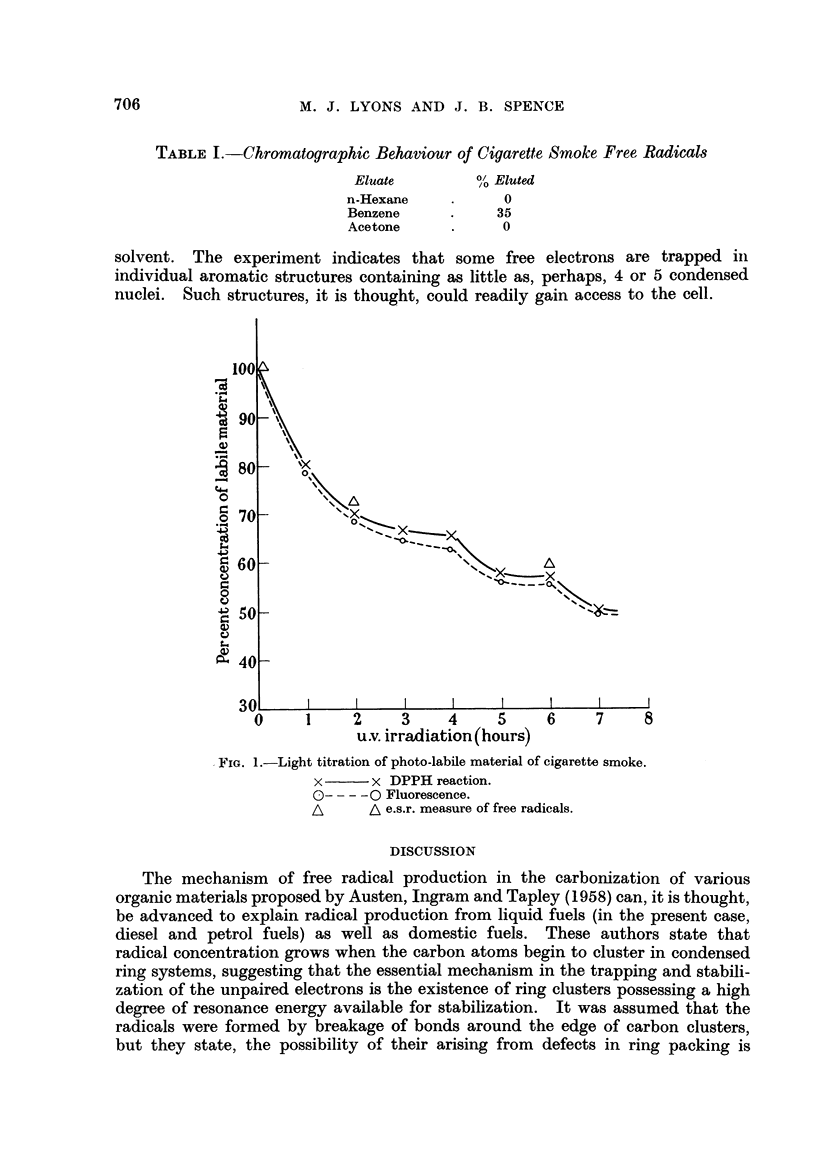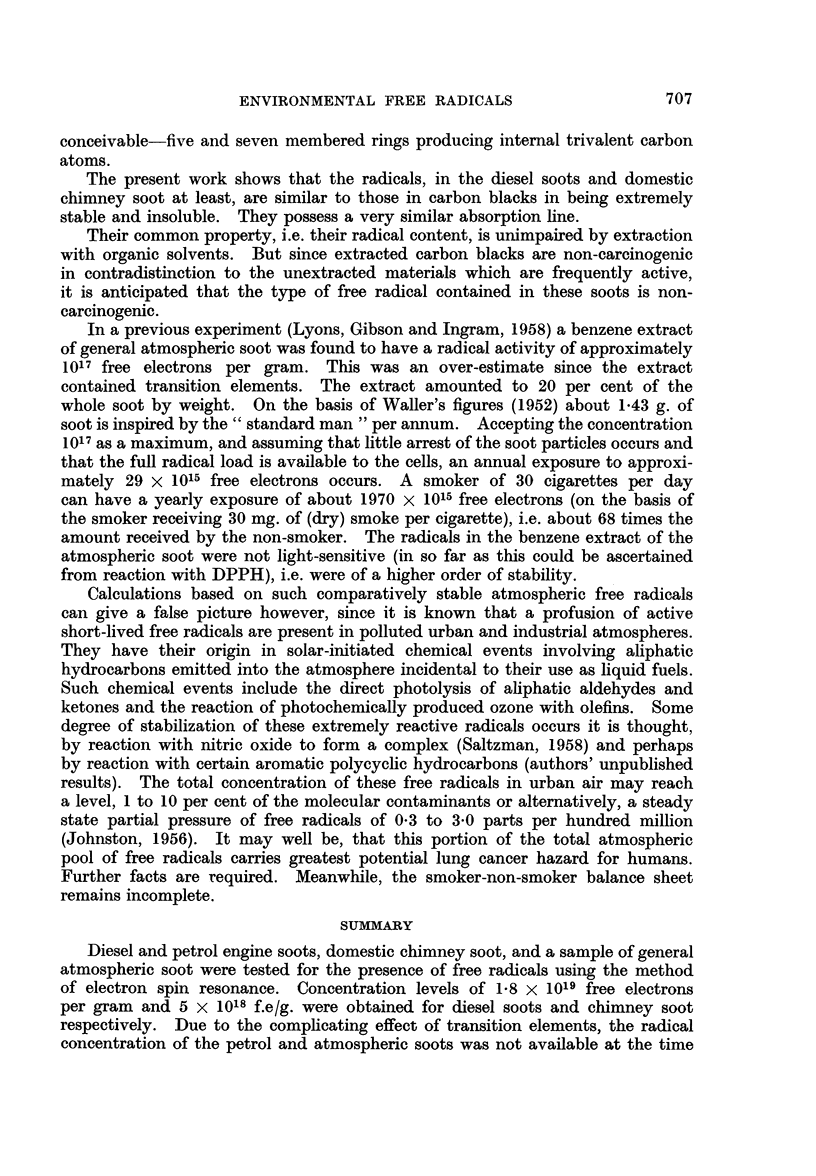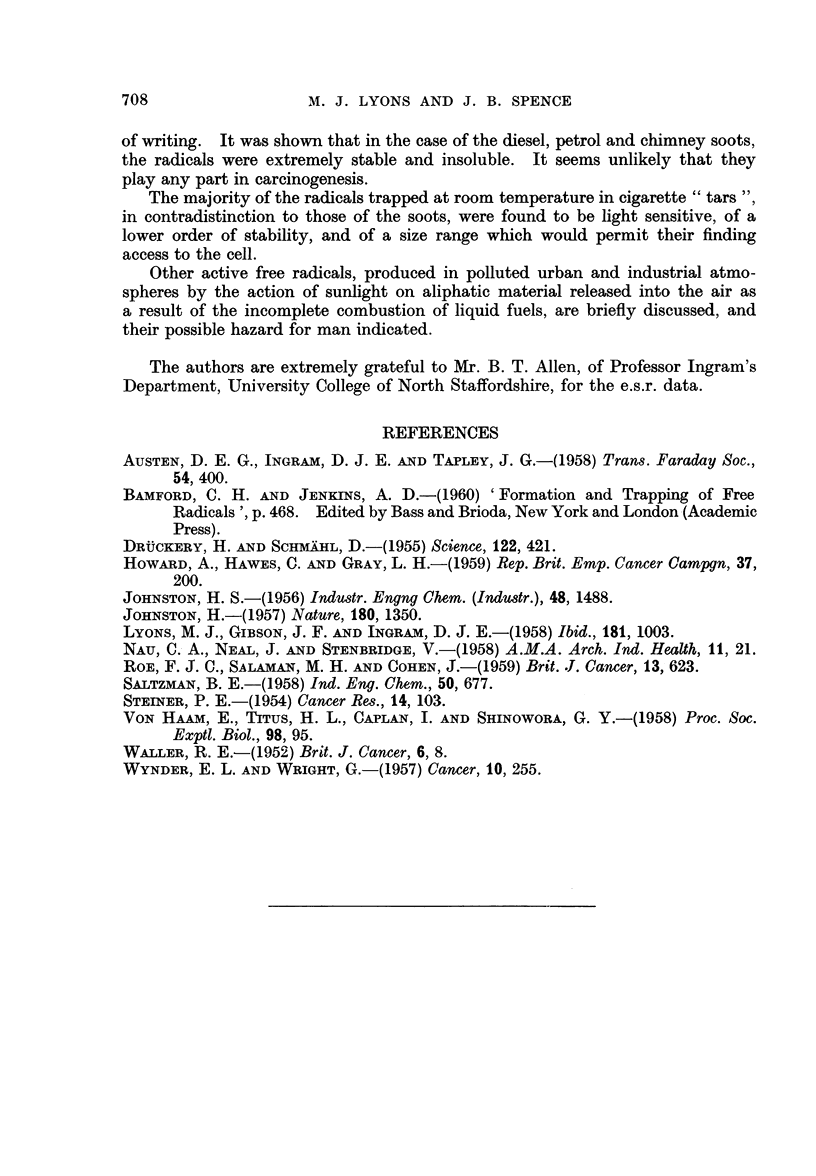# Environmental Free Radicals

**DOI:** 10.1038/bjc.1960.79

**Published:** 1960-12

**Authors:** M. J. Lyons, J. B. Spence


### ENVIRONMENTAL FREE RADICALS

M. J. LYONS* AND J. B. SPENCE

*Cancer Research Department, Royal Beatson Memorial Hospital, Glasgow*

Received for publication October 14, 1960

IN work related to the causation of human lung cancer, some investigators, appreciating the proposed special cancer environment of the cigarette smoker, seek to support this proposition by discovery of new carcinogenic agents or adjuvant factors in cigarette smoke, to which non-smokers are not especially exposed. Thus smoke phenols (Roe, Salaman and Cohen, 1959) and bases (Wynder and Wright, 1957) have been cited as co-factors in what would essentially be carcinogenesis by smoke polycyclic hydrocarbons. The discovery of appreciable quantities of free radicals in cigarette smoke (Lyons, Gibson and Ingram, 1958) led to the proposal of their possible involvement in the carcinogenic process. This followed from various suggestions in the literature on the possible pivotal role of free radical forms in cancer production by a variety of agents (for reference see Bamford and Jenkins, 1960). A more speculative aspect, perhaps, of the free radical hypothesis is the possible sensitization of cells to the deleterious action of carcinogenic agents by free radicals through their paramagnetism, an idea related to that of Howard, Hawes and Gray (1959) who are seeking to establish whether the well-known radiosensitizing action of oxygen and nitric oxide is due to the paramagnetic property of these agents.

For present purposes it was decided to attempt an estimation of the relative amounts of free radicals to which cigarette smokers and non-smokers are liable to be exposed, and to investigate any qualitative differences among such radicals which might have a bearing on their possible biological action. As regards the latter point, it seemed desirable to have an estimate of their size and stability. For these ends, the following experimental work was undertaken.

### EXPERIMENTAL

### *Free Radicals in general atmospheric pollution*

Suspected sources of general atmospheric free radicals examined were: domestic chimney smoke, vehicular exhausts and cigarette side-stream smoke.

Samples of particulate exhaust products from mechanically sound petrol and diesel enges were collected in glass containers which were kept cool during the sampling. The diesel soot samples were collected at speeds of 2080, 1400 and 800 r.p.m., at 10 per cent overload from vehicle engines on a test bed. (Negligible quantities of soot were produced at reduced loads.) The petrol engine soots were collected during acceleration-deceleration and idling periods from privately owned cars. All the exhaust condensates were filtered and dried over phosphorus pentoxide. These samples were tested for the presence of free

* Now at Section of Epidemiology, Division of Preventive Medicine. Sloan-Kettering Institute, New York, New York.

radicals by the electron spin resonance (e.s.r.) method, as were samples of domestic chimney soot and general atmospheric soot—the latter collected over a two-month period in autumn in a ventilator shaft at a site in central Glasgow.

In view of the well-known conditional carcinogenicity of soot-borne carcinogens (Steiner, 1954 ; Nau, Neal and Stembridge, 1958 ; Von Haam, Titus, Caplan and Shinowora, 1958) it was of interest to know the proportion of soot radicals likely to be available to the cells of the respiratory tract—as far as this can be known by extraction with organic solvents. Consequently, the radical levels of the soots were re-determined following the extraction of aliquots with hexane, benzene and acetone. The results were as follows :

(1) The domestic chimney soots contain $5 \times 10^{18}$ free electrons per gram of soot. This concentration was not affected by the extracting solvents, hexane, benzene and acetone, which removed 6·6, 27·5 and 41·0 per cent by weight of material, respectively.

(2) The diesel soots contained $1{\cdot}8 \times 10^{19}$ free electrons per gram, irrespective of running condition. This level was also unaffected by the extractions, which resulted in a loss by weight of 2, 6 and 7 per cent.

(3) Both petrol exhaust samples exhibited an extremely large broad resonance signal (of the order of 1000 gauss) which was unaffected by any of the solvents used.

(4) The ventilator soot also exhibited a large broad resonance.

In the case of the petrol and ventilator soots it has been suggested (B. T. Allen, personal communication) that the origin of the large broad resonance may lie in the presence of lead and iron in the samples. The free electron concentrations were not available at the time of writing. The domestic soot samples exhibit normal free radical absorption similar to carbon blacks, while the diesel soot absorption is broader, although still centred around $g = 2$. The broader absorption signal of the diesel soot relative to the domestic soot is related to its formation at a higher temperature, and is due to the interaction of the free electrons with electrons in the conduction bands (Austen, Ingram and Tapley, 1958).

Cigarette side-stream smoke was found to contain approximately $5 \times 10^{14}$ free electrons per gram. Main-stream smoke had a " stable " radical concentration about twice as high.

*Free radicals in cigarette main-stream smoke*

Cigarette main-stream smoke, condensed at liquid oxygen temperatures showed the presence of $6 \times 10^{15}$ free electrons per gram. On warming to room temperature or above, or trapping the smoke in benzene at room temperature a residual concentration of about $10^{15}$ was found. A majority of these were found to be light sensitive, as the following experiments indicate.

Drückrey and Schmähl (1955) showed that the fluorescence intensity of freshly prepared benzene solutions of cigarette smoke decreased on exposure to light. Johnston (1957) showed that the labile fluorescent components were products of combustion of the tobacco ; that similar labile material could be produced by combusting other vegetable matter ; that the percentage of the total fluorescent material which was labile varied for the various material combusted.

In the present investigation it was found that the reactivity of fresh benzene solutions of cigarette smoke with the stable free radical scavenger $\alpha\alpha'$-diphenyl-

$\beta$-picrylhydrazyl (DPPH) decreased on exposing the former to light. This decrease was found to occur even when the tobacco was extracted with acid, alkali and organic solvents prior to smoking. DPPH-reacting polar constituents of the smoke were found not to be light sensitive. It was decided to test whether the decrease in fluorescence intensity and in DPPH-activity were related phenomena and if so, to attempt an explanation.

DPPH-reacting polar reducing substances were extracted from the cigarettes prior to smoking by refluxing with dilute alkali, acid and water. After conditioning the tobacco to about a 10 per cent water content the tobacco was smoked in cigarette form. The smoke from one such cigarette in 250 ml. of benzene afforded a convenient solution for both fluorescence and DPPH-activity measurements.

Solutions were irradiated at a distance of 25 cm. with an unfiltered high pressure 125 W mercury vapour lamp, giving a band spectrum, and samples withdrawn at hourly intervals for measurement. Fluorescence measurements were made on a Uvispek spectrophotometer with a H730 fluorescence attachment, which afforded excitation at 365 m$\mu$. An 0·1 mg. per cent quinine sulphate in 0·1 N . $H_2SO_4$ standard was used. DPPH-activity was estimated from observing the rate of decrease of the compound's 520 m$\mu$ absorption maximum following the addition of 0·1 ml. of a $10^{-2}$ M solution to 10 ml. of the smoke solution. The percentage decrease in both cases was correlated with concentration by reference to dilution curves for the same solution.

A remarkably close correspondence between the decrease in fluorescence intensity and DPPH-activity was found. This is shown in Fig. 1.

An analysis of the curves shows them to be discontinuous at two points, the four- and six-hour irradiation points. A decrease of about 60 per cent in the concentration of labile components occurred in 24 hours, the major portion (50 per cent) occurring in the first seven hours of irradiation. The reaction had first order kinetics, and three different rate constants revealed the presence of three different components. Electron spin resonance measurements of a smoke solution irradiated under the same conditions for two and six hours showed that decreases in free radical concentration of 26 and 40 per cent respectively had occurred.

The light sensitive components of cigarette smoke appeared therefore to be free radicals, a light titration of which revealed the presence of at least three species of different stability.

In order to obtain some estimate of the size of such radicals, a concentrated benzene solution of smoke was added to fresh alumina (Spence, 100–200 mesh), and the solvent allowed to evaporate off in the air. The resulting alumina with adsorbed tar was filled into a column. Care was taken throughout to shield the tar from diffuse daylight. The column was washed with $n$-hexane until no further material was being eluted, and the same procedure carried out with benzene and acetone consecutively. The solvents were evaporated from the three eluates and the free radical content of the resulting tars estimated by e.s.r. The results are shown in Table I.

No radicals were detected in the hexane soluble fraction while the subsequent benzene and acetone eluates contained 35 per cent and 50 per cent of the radicals present in the original tar. Thus about 85 per cent of the radicals could be desorbed from the alumina with a facility which increased with the polarity of the

TABLE I.—*Chromatographic Behaviour of Cigarette Smoke Free Radicals*

| *Eluate* | *% Eluted* |
|---|---|
| n-Hexane . | 0 |
| Benzene . | 35 |
| Acetone . | 0 |

solvent. The experiment indicates that some free electrons are trapped in individual aromatic structures containing as little as, perhaps, 4 or 5 condensed nuclei. Such structures, it is thought, could readily gain access to the cell.

FIG. 1.—Light titration of photo-labile material of cigarette smoke.
×———× DPPH reaction.
○- - - -○ Fluorescence.
△ △ e.s.r. measure of free radicals.

DISCUSSION

The mechanism of free radical production in the carbonization of various organic materials proposed by Austen, Ingram and Tapley (1958) can, it is thought, be advanced to explain radical production from liquid fuels (in the present case, diesel and petrol fuels) as well as domestic fuels. These authors state that radical concentration grows when the carbon atoms begin to cluster in condensed ring systems, suggesting that the essential mechanism in the trapping and stabilization of the unpaired electrons is the existence of ring clusters possessing a high degree of resonance energy available for stabilization. It was assumed that the radicals were formed by breakage of bonds around the edge of carbon clusters, but they state, the possibility of their arising from defects in ring packing is conceivable—five and seven membered rings producing internal trivalent carbon atoms.

The present work shows that the radicals, in the diesel soots and domestic chimney soot at least, are similar to those in carbon blacks in being extremely stable and insoluble. They possess a very similar absorption line.

Their common property, i.e. their radical content, is unimpaired by extraction with organic solvents. But since extracted carbon blacks are non-carcinogenic in contradistinction to the unextracted materials which are frequently active, it is anticipated that the type of free radical contained in these soots is non-carcinogenic.

In a previous experiment (Lyons, Gibson and Ingram, 1958) a benzene extract of general atmospheric soot was found to have a radical activity of approximately $10^{17}$ free electrons per gram. This was an over-estimate since the extract contained transition elements. The extract amounted to 20 per cent of the whole soot by weight. On the basis of Waller's figures (1952) about 1·43 g. of soot is inspired by the " standard man " per annum. Accepting the concentration $10^{17}$ as a maximum, and assuming that little arrest of the soot particles occurs and that the full radical load is available to the cells, an annual exposure to approximately $29 \times 10^{15}$ free electrons occurs. A smoker of 30 cigarettes per day can have a yearly exposure of about $1970 \times 10^{15}$ free electrons (on the basis of the smoker receiving 30 mg. of (dry) smoke per cigarette), i.e. about 68 times the amount received by the non-smoker. The radicals in the benzene extract of the atmospheric soot were not light-sensitive (in so far as this could be ascertained from reaction with DPPH), i.e. were of a higher order of stability.

Calculations based on such comparatively stable atmospheric free radicals can give a false picture however, since it is known that a profusion of active short-lived free radicals are present in polluted urban and industrial atmospheres. They have their origin in solar-initiated chemical events involving aliphatic hydrocarbons emitted into the atmosphere incidental to their use as liquid fuels. Such chemical events include the direct photolysis of aliphatic aldehydes and ketones and the reaction of photochemically produced ozone with olefins. Some degree of stabilization of these extremely reactive radicals occurs it is thought, by reaction with nitric oxide to form a complex (Saltzman, 1958) and perhaps by reaction with certain aromatic polycyclic hydrocarbons (authors' unpublished results). The total concentration of these free radicals in urban air may reach a level, 1 to 10 per cent of the molecular contaminants or alternatively, a steady state partial pressure of free radicals of 0·3 to 3·0 parts per hundred million (Johnston, 1956). It may well be, that this portion of the total atmospheric pool of free radicals carries greatest potential lung cancer hazard for humans. Further facts are required. Meanwhile, the smoker-non-smoker balance sheet remains incomplete.

### SUMMARY

Diesel and petrol engine soots, domestic chimney soot, and a sample of general atmospheric soot were tested for the presence of free radicals using the method of electron spin resonance. Concentration levels of $1 \cdot 8 \times 10^{19}$ free electrons per gram and $5 \times 10^{18}$ f.e/g. were obtained for diesel soots and chimney soot respectively. Due to the complicating effect of transition elements, the radical concentration of the petrol and atmospheric soots was not available at the time of writing. It was shown that in the case of the diesel, petrol and chimney soots, the radicals were extremely stable and insoluble. It seems unlikely that they play any part in carcinogenesis.

The majority of the radicals trapped at room temperature in cigarette " tars ", in contradistinction to those of the soots, were found to be light sensitive, of a lower order of stability, and of a size range which would permit their finding access to the cell.

Other active free radicals, produced in polluted urban and industrial atmospheres by the action of sunlight on aliphatic material released into the air as a result of the incomplete combustion of liquid fuels, are briefly discussed, and their possible hazard for man indicated.

The authors are extremely grateful to Mr. B. T. Allen, of Professor Ingram's Department, University College of North Staffordshire, for the e.s.r. data.

### REFERENCES

AUSTEN, D. E. G., INGRAM, D. J. E. AND TAPLEY, J. G.—(1958) *Trans. Faraday Soc.*, **54**, 400.

BAMFORD, C. H. AND JENKINS, A. D.—(1960) 'Formation and Trapping of Free Radicals', p. 468. Edited by Bass and Brioda, New York and London (Academic Press).

DRÜCKERY, H. AND SCHMÄHL, D.—(1955) *Science*, **122**, 421.

HOWARD, A., HAWES, C. AND GRAY, L. H.—(1959) *Rep. Brit. Emp. Cancer Campgn*, **37**, 200.

JOHNSTON, H. S.—(1956) *Industr. Engng Chem. (Industr.)*, **48**, 1488.

JOHNSTON, H.—(1957) *Nature*, **180**, 1350.

LYONS, M. J., GIBSON, J. F. AND INGRAM, D. J. E.—(1958) *Ibid.*, **181**, 1003.

NAU, C. A., NEAL, J. AND STENBRIDGE, V.—(1958) *A.M.A. Arch. Ind. Health*, **11**, 21.

ROE, F. J. C., SALAMAN, M. H. AND COHEN, J.—(1959) *Brit. J. Cancer*, **13**, 623.

SALTZMAN, B. E.—(1958) *Ind. Eng. Chem.*, **50**, 677.

STEINER, P. E.—(1954) *Cancer Res.*, **14**, 103.

VON HAAM, E., TITUS, H. L., CAPLAN, I. AND SHINOWORA, G. Y.—(1958) *Proc. Soc. Exptl. Biol.*, **98**, 95.

WALLER, R. E.—(1952) *Brit. J. Cancer*, **6**, 8.

WYNDER, E. L. AND WRIGHT, G.—(1957) *Cancer*, **10**, 255.